# Role of Interleukin-17A in the Pathomechanisms of Periodontitis and Related Systemic Chronic Inflammatory Diseases

**DOI:** 10.3389/fimmu.2022.862415

**Published:** 2022-03-17

**Authors:** Yi Feng, Zheng Chen, Shao-Qin Tu, Jia-Ming Wei, Yu-Luan Hou, Zhi-Li Kuang, Xiao-Ning Kang, Hong Ai

**Affiliations:** Department of Stomatology, The Third Affiliated Hospital of Sun Yat-sen University, Guangzhou, China

**Keywords:** IL-17A, inflammation, periodontitis, chronic inflammatory diseases, rheumatoid arthritis, psoriasis

## Abstract

Periodontitis is a chronic inflammatory and destructive disease caused by periodontal microbial infection and mediated by host immune response. As the main cause of loosening and loss of teeth in adults, it is considered to be one of the most common and serious oral diseases in the world. The co-existence of periodontitis and systemic chronic inflammatory diseases such as rheumatoid arthritis, psoriasis, inflammatory bowel disease, diabetes and so on is very common. It has been found that interleukin-17A (IL-17A) secreted by various innate and adaptive immune cells can activate a series of inflammatory cascade reactions, which mediates the occurrence and development of periodontitis and related systemic chronic inflammatory diseases. In this work, we review the role of IL-17A in the pathomechanisms of periodontitis and related systemic chronic inflammatory diseases, and briefly discuss the therapeutic potential of cytokine targeted agents that modulate the IL-17A signaling. A deep understanding of the possible molecular mechanisms in the relationship between periodontitis and systemic diseases will help dentists and physicians update their clinical diagnosis and treatment ideas.

## Introduction

Immune response is divided into innate immunity and adaptive immunity. Innate immunity is the first line of defense against pathogen invasion. Cells involved in innate immunity include monocytes/macrophages, dendritic cells (DC), granulocytes, natural killer (NK) cells and NKT cells, which can recognize the pathogen associated molecular patterns expressed by pathogenic organisms. For example, lipopolysaccharide, an outer membrane component of Gram-negative bacteria including *Porphyromonas gingivalis* (one of the pathogens of periodontitis), can be recognized by toll like receptor 4 (TLR-4) on the surface of monocytes/macrophages or DC, resulting in innate immune response. Adaptive immunity includes humoral immunity and cell-mediated immunity, which are mediated by B cells and T cells respectively. B cells produce antibodies against extracellular pathogens and toxins, while T cells mediate cellular immunity against intracellular pathogens. Innate immunity is closely related to adaptive immunity. Innate immunity is the initiating factor of adaptive immunity and can provide the activation signals required by adaptive immune response. Effector molecules of adaptive immunity can also greatly promote the innate immune response, such as IFN-γ secreted by helper T (Th) cells strongly activate macrophages and NK cells, enhancing their phagocytosis and killing function.

In the process of T cell-mediated cellular immunity, after the naive CD4 (+) T cells and CD8 (+) T cells are activated, they proliferate and differentiate into effector cells under the cytokines and other factors in the local micro-environment, and form different functional subsets including Th cells and cytotoxic T lymphocytes (CTL) which play auxiliary functions or follow the blood circulation to the specific antigen site to play effector functions respectively. IL-12 and IFN- γ can induce the differentiation of naive CD4 (+) T cells into Th1 cells which mainly mediate the cellular immune response. IL-4 can induce naive CD4 (+) T cells to differentiate into Th2 cells which mainly mediate the humoral immune response. IL-2 and TGF-β can induce naive CD4 (+) T cells to differentiate into regulatory T cells (Tregs) which play a negative immunosuppressive function by secretion of cytokines or cell contact and play an important role in maintaining autoimmune tolerance. IL-1 β, IL-6, and IL-23 participate in the differentiation from Th0 cells to Th17 cells. The main biological function of Th17 cells is to mediate the occurrence and development of several inflammatory responses and autoimmune diseases. Th1, Th2 and Th17 cells secrete cytokines of different lineages. Th1 cells mainly secrete IL-2 and IFN- γ, whose effector cells include CD4 (+) T cells, CTL, macrophages and part of B cells (1). Th2 cells mainly secrete IL-4 and IL-5, which mainly act directly on B cells (1). The occurrence and development of periodontitis is related to the breaking of the fine balance between Th1 and T2 cytokines (2). Th17 cells mainly secrete IL-17 and IL-22. Among them, IL-17A can act on a variety of different effector cells, such as neutrophils, NK cells, macrophages, epithelial cells, endothelial cells and osteoblasts. It plays a key role in the host defense against infection. At the same time, it also plays an important role in the occurrence and development of chronic inflammatory diseases such as rheumatoid arthritis, psoriasis, inflammatory bowel disease, diabetes, and periodontitis (3). Among them, periodontitis is considered as an independent risk factor for rheumatoid arthritis, diabetes, osteoporosis, atherosclerosis and other systemic diseases (4–6).

**Figure 1:**
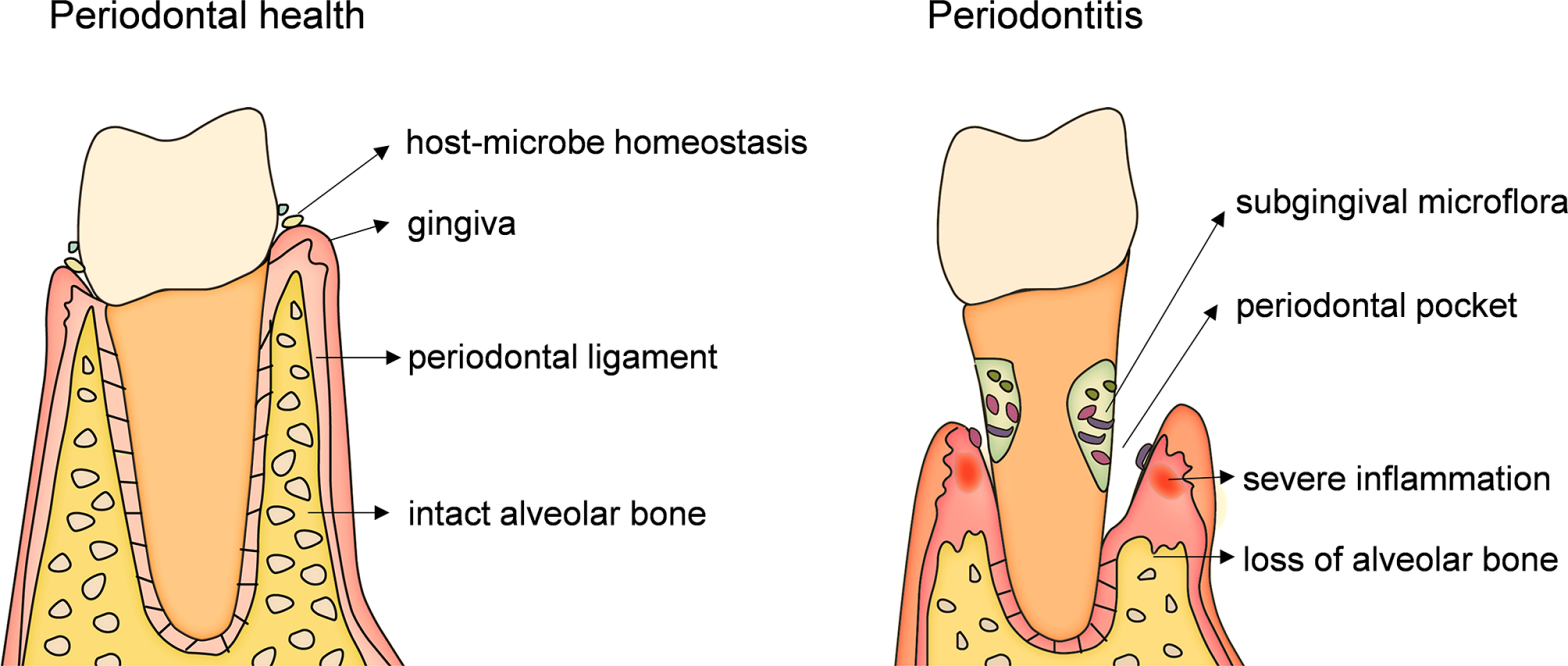
An anatomical illustration of periodontitis. caused by infection of microbial from dental plaque biofilm. Pathogenic microorganisms and their products act on gingiva for a long time and cause immune response which first leads to gingival inflammation. When inflammation spreads to the deep periodontal tissue, causing the dissolution and destruction of collagen fibers in the gingiva and periodontal ligament and absorption of alveolar bone, resulting in the formation of periodontal pocket, which is the occurrence of periodontitis. The main symptoms of periodontitis are inflammation and bleeding of gums, formation of periodontal pockets, loss of alveolar bone, loosening and loss of teeth.

Periodontitis, the main cause of loosening and loss of teeth in adults, is a chronic inflammatory and destructive disease caused by periodontal microbial infection and mediated by host immune response. It is typically characterized by inflammatory infiltration of periodontal supporting tissues (gingiva, periodontal ligament, alveolar bone and cementum) and damage and loss of alveolar bone (**Figure 1**). Periodontitis is currently one of the most common and serious oral diseases in the world and severe periodontitis is the sixth most prevalent health condition (7, 8). The antigen components, enzymes, toxins and metabolites of periodontal pathogens can directly destroy the periodontal tissues, or cause local immune and inflammatory reactions, resulting in periodontal tissues damage. Although the periodontal tissues also have the host congenital immune defense system against periodontal pathogens, and responds to the adhesion and invasion of bacteria into the tissues through the barrier structure with physical, chemical and biological effects. The ability of the pathogenic bacteria to escape and destroy the innate immune response, as well as the defects and disorders of the host’s own innate immune mechanism, lead to the further spread of infection and inflammation. The innate immune response of the host enters the induction stage of adaptive immune response. The protective - destructive mechanism of the host immunity leads to local periodontal tissues damage and promotes the process of periodontitis.

The co-existence of periodontitis and systemic chronic inflammatory diseases is very common (3). For example, diabetes mellitus has been proved to be one of the risk factors of periodontitis, and periodontitis is classified as the sixth complication of diabetes. What’s more, severe periodontitis is highly correlated with poor blood glucose control (9), suggesting that there is a bidirectional interaction between periodontitis and related chronic inflammatory diseases. Although the relationship between periodontitis and these diseases and their pathological mechanisms have not been elucidated and their etiologies are also different, there are persistent immune inflammatory responses and elevated levels of inflammatory mediators in the internal environment. Therefore, revealing the complex cytokine signal networks will provide new ideas for the diagnosis and treatment of periodontitis and related systemic chronic inflammatory diseases. It has been found that interleukin-17A (IL-17A) secreted by a variety of innate immune cells and adaptive immune cells including Th17 cells can activate a series of inflammation related cascade reactions and mediate the occurrence and development of periodontitis and related systemic chronic inflammatory diseases. This work aims to provide an overview of the role of IL-17A in the pathomechanisms of periodontitis and related systemic chronic inflammatory diseases and briefly discuss the therapeutic potential of cytokine targeted agents that modulate the IL-17A signaling. A deep understanding of the possible molecular mechanisms in the relationship between periodontitis and systemic diseases will help dentists and physicians update their clinical diagnosis and treatment ideas, so as to formulate more reasonable diagnostic methods, preventive measures and treatment strategies.

## The Il-17 Family and the Cellular Sources of Il-17a

In 1993, Rouvier et al. (10) discovered IL-17A cDNA in the process of screening lymphocyte gene expression library to explore new molecules related to immune function. At that time, it was named cytotoxic T lymphocyte associated antigen 8 (CTLA8) and was considered to be related to the immune system. In 1995, Yao et al. (11) isolated a new receptor encoded by cDNA binding CTLA8, and suggested that CTLA8 and its receptor be named IL-17 (now IL-17A) and IL-17R respectively. Currently, the IL-17 family has six members, including IL-17A, IL-17B, IL-17C, IL-17D, IL-17E (IL-25) and IL-17F. In addition to IL17D, all members play biological functions in the form of homodimer. IL-17A and IL-17F can also form heterodimer (12, 13). There are five members in the IL-17 family of receptors, including IL-17RA, IL-17RB/IL-25R, IL-17RC, IL-17RD/SEF and IL-17RE (**Table 1**). The main functions of IL-17A include inducing host defense response to extracellular bacteria and fungi and mediating the occurrence and development of chronic immune-mediated inflammatory diseases (19). IL-17E plays an important role in the host’s defense against parasites and the progression of virus-related asthma, and IL-17B can inhibit the inflammatory process mediated by IL-17E (16). IL-17C is not produced by immune cells but by epithelial cells and keratinocytes, which can maintain the stability of skin and the intestinal barrier (17). IL-17D-CD93 axis maintains intestinal homeostasis by regulating the function of group 3 innate lymphoid cells (ILC3s) (18).

**Table 1:** The IL-17 family and its receptors.

IL-17-family ligands	IL-17 receptors (IL-17R)
IL-17A homodimer (IL-17A/A)	IL-17RA and IL-17RC (IL-17RA/RC) (14), IL17RC/RC (14), IL17RA/RD (15)
IL-17A/F heterodimer	IL-17RA/RC, IL17RC/RC (14)
IL-17F/F homodimer	IL-17RA/RC, IL17RC/RC (14)
IL-17B/B	IL-17RA/RB (16)
IL-17C/C	IL-17RA/RE (17)
IL-17D	CD93 (18)
IL-17E/E	IL-17RA/RB (16)

The traditional view is that the biological effects of IL-17F and IL-17A are the same because they have the same receptors. Ishigame et al. showed that there are differences in the biological functions of IL-17F and IL-17A, and the cytokine profiles of macrophages and T cells stimulated by IL-17A and IL-17F respectively are different (20). The production of IL-17 family and its different roles and regulatory mechanisms in the immune system remain to be elucidated.

IL-17A is the most deeply studied. IL-17A is a characteristic cytokine secreted by Th17 cells, a distinct CD4 (+) T cell subset, known as T helper 17 cells (Th17). However, in addition to Th17 cells, a variety of innate and adaptive immune cells including NKT cells, mucosal associated invariant T (MAIT) cells, ILC3s, neutral regulatory T cells (Tn cells), γδ T cells and Tc17 cells can secrete IL-17A (21–26). Different from Th17 cells, Tc17 is a subset of CD8 (+) T cells. The obvious difference between Tc17 cells and the classical subsets of CD8 (+) T cells (Tc1 and Tc2 cells) is that Tc17 cells lack cytotoxicity and are characterized by secretion of IL-17A. They play an important role in the occurrence and development of psoriasis, rheumatoid arthritis, diabetes mellitus and other diseases (26–28). ILC3s can be activated by surface activation related receptors that are stimulated by IL-1β and IL-23 produced by macrophages or dendritic cells infected by extracellular pathogens, and secrete IL-22 and IL-17 to participate in anti-extracellular bacterial/fungal infection or the intestinal inflammation (23, 29). γδ T cells have been confirmed to be the main source of IL-17A in Listeria monocytogenes and Escherichia coli infection (30, 31). In addition, γδ T cells/IL-17A axis is also closely related to diseases such as psoriasis and bronchial asthma (25, 32).

## Il-17A Signalling

IL-17A acts on different effector cells through various signal transduction pathways and cascade reactions, resulting in different physiological or pathological effects. IL-17A homodimer and heterodimer with IL-17F (IL-17A, IL-17F, and IL-17A/F heterodimer) can bind to receptors including IL-17RA and IL-17RC. Act1 binds to IL-17RA and IL-17RC through the SEFIR domain of them (33), and then recruits TRAF6 (34). TRAF6 then activates TGF-β-activated kinase (TAK1) to activate NF- κ B, JNK, p38-AP-1, ERK1/2-C/EBP δ, C/EBP δ (35). TRAF3 and TRAF4 negatively regulate the IL-17A signaling pathway. TRAF3 inhibits the binding of Act1 to IL-17R, while TRAF4 inhibits the recruitment of TRAF6 by Act1 (36, 37).

Recent studies have found that IL-17RD, which directly binds to IL-17A and IL-17RA to form heterodimer but not to IL-17F or IL-17A/F heterodimer, is a functional receptor of IL-17A. It plays an important role in the pathogenesis of psoriasis like skin inflammation (15). However, the specific molecular mechanism of signal transduction has not been fully clarified. In addition, the efficiency of IL-17RA binding IL-17A is higher than that of IL-17 ~ 1000-fold higher affinity (38).

## Il-17A In Health and Disease

**Figure 2:**
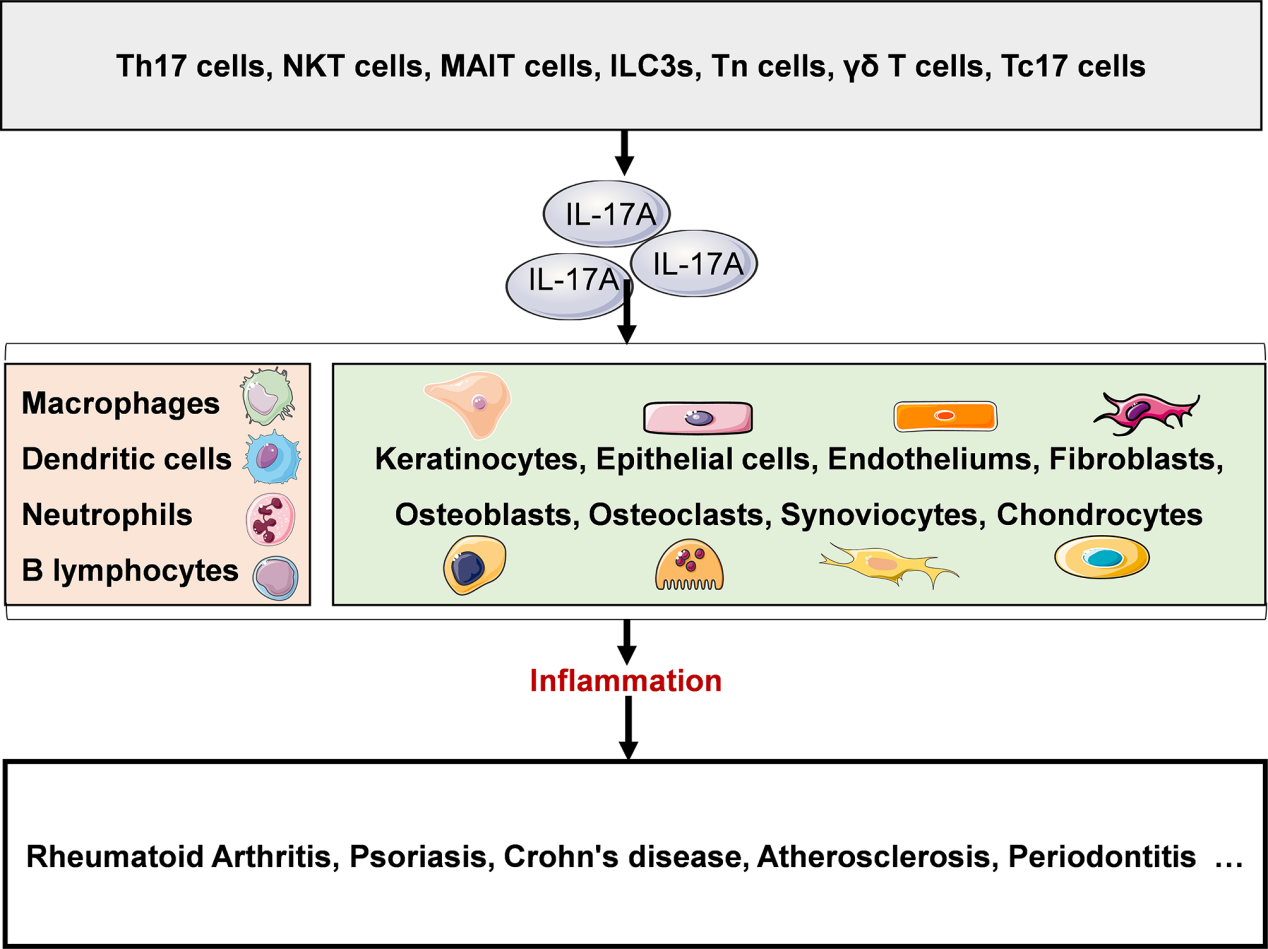
IL-17A in disease. A variety of innate and adaptive immune cells include (NKT cells, MAIT cells, ILC3s, Tn cells, γδ T cells, Tc17 cells and Th17 cells can secrete IL-17A. IL-17A plays a proinflammatory role and is closely associated with several chronic inflammatory diseases, such as rheumatoid arthritis, psoriasis, Crohn’s disease, atherosclerosis, and periodontitis. NKT, natural killer T; MAIT, mucosal associated invariant T; ILC3s, group 3 innate lymphoid cells; Tn, naive T; γδ T, gamma-delta T; Th, helper T. Original elements used in this diagram and **Figures 3**–**5** are from Servier Medical Art (smart.servier.com/), and were modified under a Creative Common Attribution 3.0 Generic License (creativecommons.org/lic…).

IL-17A plays a key role in both physiological and pathological states. Under physiological conditions, IL-17A is produced rapidly in response to microbial infections such as bacteria, fungi and viruses, and plays an anti-infection role. Under pathological conditions such as chronic inflammatory diseases, IL-17A plays a proinflammatory role, mediates pathological inflammatory response, and promotes the occurrence, development and chronicity of diseases. At present, IL-17A related diseases have been studied, including rheumatoid arthritis, psoriasis, Crohn’s disease, atherosclerosis, periodontitis and so on (**Figure 2**).

IL-17A can activate immune cells such as B cells and macrophages, thereby promoting antibody production and expression of pro-inflammatory factors (IL-1, IL-6, TNF, G-CSF, and GM-CSF) (39), and activating epithelial cells, fibroblasts, endothelial cells and other non-immune cells to induce antimicrobial peptides and a variety of pro-inflammatory mediators including cytokines, chemokines (CXCL1, CXCL5, IL-8, CCL2 and CCL7), MMPs, VEGF and RANKL, which play a role in recruiting neutrophils, mediating local tissue destruction, inducing tumor neovascularization and promoting osteoclast formation, so as to achieve host defense and promote progression of diseases (40, 41).

IL-17F has similar effects, but its ability to induce the expression of proinflammatory factors is weaker (42). Current studies have shown that IL-17A is mainly closely related to autoimmunity, allergic reaction, tumor progression and host defense against microbial infection, while IL-17F plays an important role in host resistance to bacteria and inflammation in epithelial tissue (42).

## Effects Of Il-17A on Immune Cells

IL-17A has a wide range of physiological effects and mediates pathological processes because it can act on many different cell types. Il-17 receptor complex exists in a variety of tissues and cells. IL-17RA is highly expressed in hematopoietic cells, while IL-17RC is mainly found in non-hematopoietic lineage (38, 43). Ishigame et al. found that both IL-17RA and IL-17RC exist in macrophages (20).

**Figure 3:**
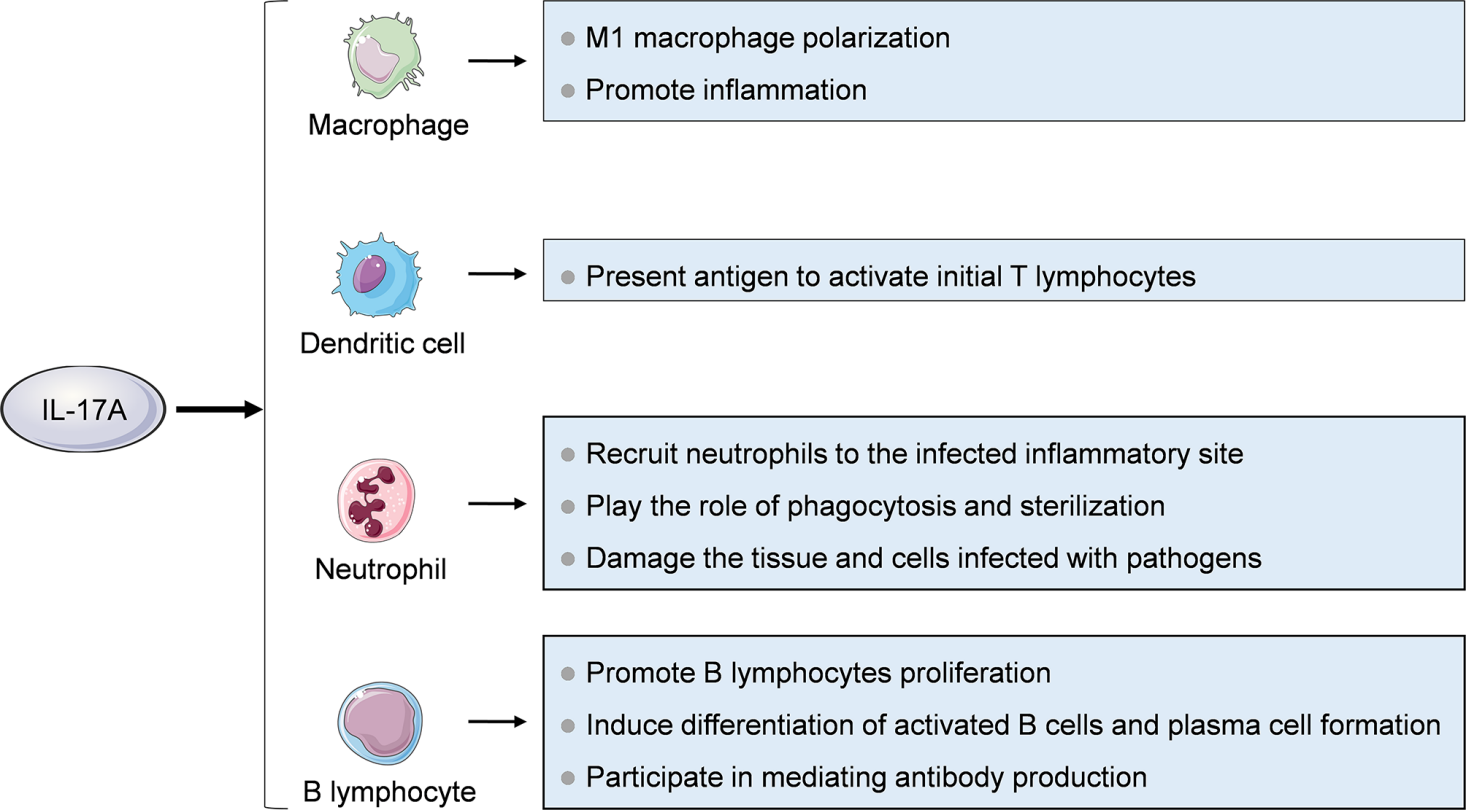
Effects of IL-17A on immune cells. Immune cells including macrophages, neutrophils, dendritic cells and B cells are cellular targets of IL-17A.

Immune cells such as macrophages, neutrophils, dendritic cells, and B cells are cellular targets of IL-17A (**Figure 3**). Macrophages are differentiated from monocytes which migrate from blood to tissues and organs under the stimulation of monocyte chemoattractant protein 1 (MCP-1) and other chemokines. Macrophages can be polarized into two subsets with distinct functional properties. M1 macrophages can eliminate pathogens by producing reactive oxygen intermediates and nitric oxide and lysosomal enzymes, and release chemokines such as CCL2, CCL3 and CXCL8 and proinflammatory factors such as IL-1 β, IL-6 and TNF-α to induce inflammation. M2 macrophages secrete cytokines such as IL-10, TGF-β, PDGF and FGF, mediating anti-inflammatory effects and participating in repair and fibrosis of damaged tissues. IL-17A can promote M1 macrophage polarization, thus exerting proinflammatory effects (44). Neutrophils are important effector cells involved in inflammation. The main role of IL-17A on neutrophils is to recruit them to the infected inflammatory site, play the role of phagocytosis and sterilization, and damage the tissue and cells infected with pathogens (45, 46). In addition, Bai et al. found that IL-17A stimulated neutrophils to release S100A8/A9 and promoted apoptosis of pulmonary epithelial cells to mediate mycoplasma pneumoniae pneumonia in children (47). Mature dendritic cells (DC) can secrete CCL18 which has chemotactic effect on naive T cells, and highly express MHC class II molecules and co-stimulatory molecules which can effectively present antigen to activate naive T cells and start adaptive immune response. It has been shown that the IL-17A treated bone marrow cell-derived DC precursor had increased expression of co-stimulatory molecules and MHC II, so its ability to improve T cell function is enhanced (48). IL-17A can also promote B cells proliferation, induce differentiation of activated B cells and plasma cell formation, and participate in mediating antibody production (42).

## Effects Of Il-17A on Other Cells

**Figure 4:**
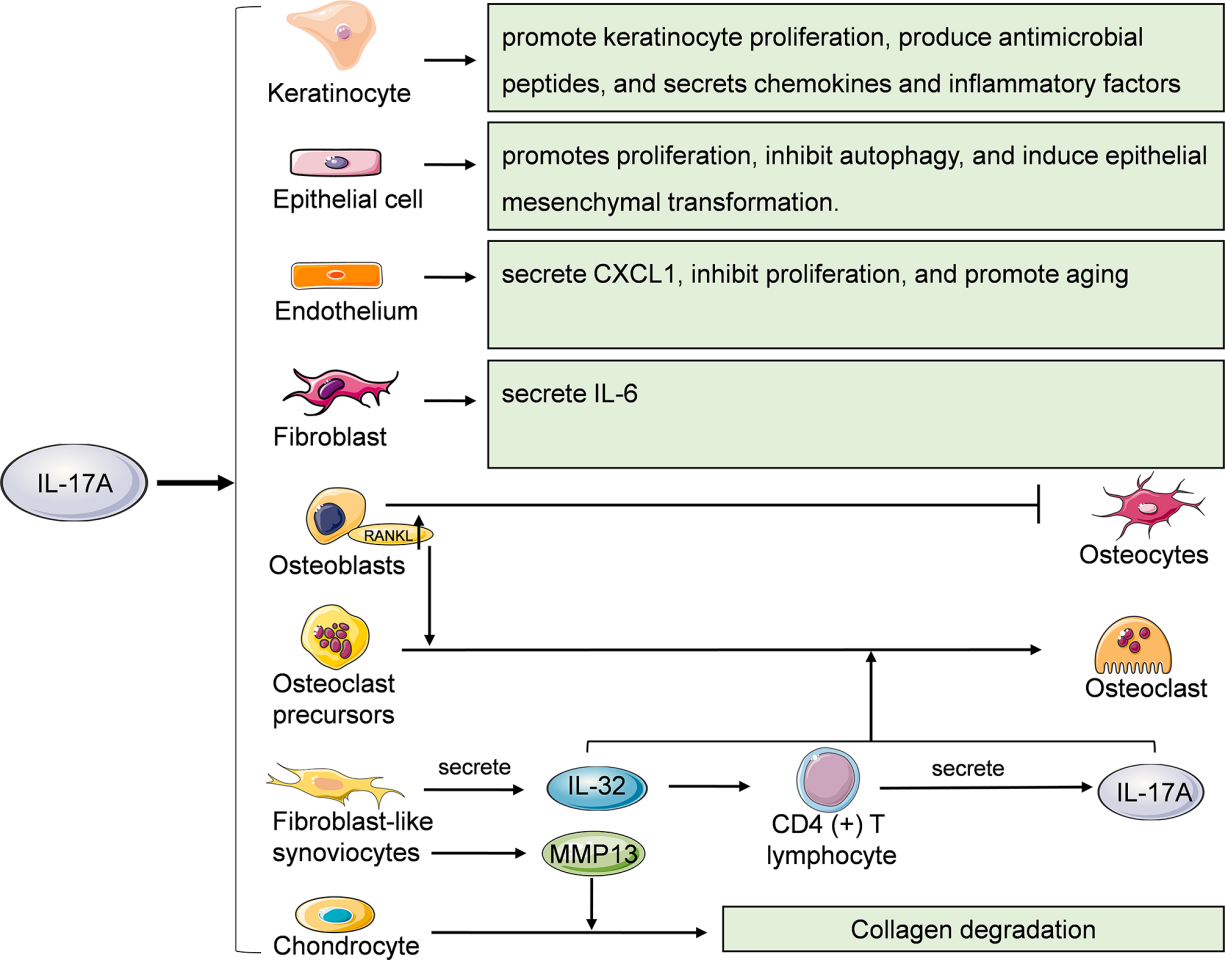
Effects of IL-17A on other cells. Keratinocytes, epithelial cells, endothelial cells, fibroblasts, osteoblasts, osteoclasts, fibroblast like synoviocytes (FLSs) and chondrocytes are also cellular targets of IL-17A. RANKL, receptor activator of nuclear factor kappa B ligand; MMP13, matrix metalloproteinase 13.

IL-17A can also act on non-immune cells such as keratinocytes, epithelial cells, endothelial cells, fibroblasts, osteoblasts, osteoclasts, fibroblast like synoviocytes (FLSs) and chondrocytes (**Figure 4**). Keratinocytes are the main cell components of the epidermis. IL-17A can promote the proliferation of keratinocytes and induce them to produce antibacterial peptides such as S100A7, S100A8, S100A9, LL-37 and DEFB4A which can enhance the local skin inflammation of the skin (49–51). IL-17A can also promote keratinocytes to secrete chemokines such as CCL20, CXCL1, CXCL2 and CXCL8 and inflammatory factors such as IL-6 and TNF-α (52). CXCL1, CXCL2, and CXCL8 can recruit neutrophils, while CCL20 can recruits CCR6 (+) IL-17A-secreting Th17 cells, ILC3, Tc17 and γδ T cells, further forming an IL-17A-rich environment (53). IL-17A is associated with the histopathological features of psoriasis, such as epidermal hyperplasia and intraepidermal neutrophil micro-abscess.

IL-17A acts on epithelial cells of various organs and tissues. IL-17A promotes the proliferation and secretion of extracellular matrix of renal tubular epithelial cells, and induces epithelial-mesenchymal transition through TGF-β1-dependent pathway, thus mediating renal fibrosis (54, 55). Fine particulate matter (PM2.5) can induces γδ T and Th17 cells secrete IL-17A to activate PI3K/AKT/mTOR signaling pathway, thus inhibiting autophagy of bronchial epithelial cells and promoting lung inflammation and fibrosis (56). IL-17A induces epithelial mesenchymal transformation of intrahepatic biliary epithelial cells, thereby promoting the occurrence and development of primary biliary cirrhosis (57).

Endothelial cells are the main cellular component of the lining of blood vessels. Among the members of IL-17 family, IL-17A, IL-17A/F heterodimer and IL-17E/IL-25 are angiogenesis stimulants, while IL-17B and IL-17F are angiogenesis inhibitors (58). Vascular endothelial cells are one of the cell types that produce CXC chemokines. IL-17A can stimulate lung microvascular endothelial cells (LMVEs) to secrete CXCL1 but not CXCL5 and CXCL8. However, IL-1β-induced LMVECs can secrete of CXCL1, CXCL5 and CXCL8, which play an important role in the activation and recruitment of neutrophils during airway inflammation (59). Recently, Slowikowski et al. found that IL-17A inhibited the proliferation and promoted senescence of murine aortic endothelial cells by activating NF-κB/p53/Rb pathway (60).

Fibroblasts are the main cellular component of loose connective tissue and participate in the tissue repair after injury. Yao et al. found that IL-17A can induce fibroblasts to secrete IL-6 and activate transcription factor NF-κB (11). However, Slowikowski et al. reported that in rheumatoid arthritis, IL-17A and TNF synergistically stimulated fibroblasts to secrete IL-6, while IL-17A alone had little effect on fibroblasts (61).

Under physiological conditions, the functions of osteoblasts and osteoclasts are in balance. The imbalance between bone formation and resorption can lead to a variety of diseases, such as primary osteoporosis, rheumatoid arthritis and so on. IL-17A can induce mRNA expression of the Wnt antagonist sFRP1 in skull osteoblasts *in vitro* and inhibit the expression of sFRP3, thus inhibiting osteoblast differentiation (62). IL-17 can promote the expression of RANKL in osteoblasts, thereby promoting RANK signal transduction in osteoclasts and mediating bone resorption (63). IL-17A can also induce osteoclastogenesis without RANKL. IL-17A can stimulate FLSs to secrete IL-32 which further induces CD4 (+) T cells to produce IL-17A in patients with rheumatoid arthritis. IL-32 and IL-17A coordinate to promote osteoclastogenesis without RANKL (64). IL-17A can also significantly up-regulate the expression level of MMP13 in FLSs, thereby inhibiting the expression of COL2A1 in chondrocyte in the co-culture system, promoting collagen degradation and mediating rheumatoid arthritis related cartilage damage (65).

## Role Of Il-17A in the Development of Periodontititis

The occurrence and development of periodontitis involves a series of immune and inflammatory reactions. Periodontal tissues damage caused by periodontitis is mainly attributing to the host’s immune response to infected microorganisms and their toxic products, not just directly caused by the infected microorganisms. The innate and adaptive immune defense and inflammatory defense that occur when the body prevents microbial invasion and diffusion will damage the local periodontal tissues. Therefore, the protective and destructive mechanism of the host immunity is an important link in the progression of periodontitis. When periodontal tissue was infected by pathogens such as *Tannerella forsythia*, *Porphyromonas gingivalis*, *Actinobacillus actinomycetemcomitans* and *Prevotella intermedia*, neutrophils and macrophages phagocytize and kill pathogens, which not only play an immune activation and regulatory role, but also promotes the local inflammatory response of periodontal tissue (66, 67). DC subsets mature and present antigens as antigen presenting cells to T cells (68). An important regulatory mechanism of Th1 and Th2 cells to host immune response is the secretion of cytokines, which can induce macrophages to secrete inflammatory factors and promote the activation and proliferation of B cells (67, 68). Activated B cells secrete antibodies while plasma cells secrete TNF-α, IL-6, IL-10, TGF-β and MMPs (69). Local infiltrating plasma cells during periodontal inflammation may be an important reason for the imbalance between MMPs and its blocker TIMPs. This lone-term chronic inflammatory response results in the absorption of alveolar bone by osteoclasts and the degradation of periodontal membrane fibers by MMPs. The ratio of Th17/Treg cells in gingival tissue and peripheral blood of patients with chronic periodontitis was significantly higher than that of healthy people (70), and the number of γδT cells in gingival tissue was also higher than that of healthy people (71). As what mentioned before, both Th17 cells and γδ T cells are the cell sources of IL-17A, suggesting that the expression level of IL-17A in periodontal region of patients with chronic periodontitis is significantly higher than that of healthy people, which has been confirmed by many studies in recent years (72–74). Furthermore, it has been reported that the expression level of IL-17A mRNA in gingival tissue of patients with chronic periodontitis was higher than that of patients with gingivitis (75).

IL-17A plays a protective and destructive role in the progression of chronic periodontitis. When periodontal infection occurs, neutrophils can quickly move out of the blood vessel and are the first effector cells that reach the infected site. IL-17A can regulate neutrophils to leave the bone marrow and enter the blood circulation, and recruit neutrophils to the infected site of periodontal tissues (76, 77). Neutrophils phagocytize bacteria and sterilize through oxygen dependent and independent mechanisms to achieve immune activation and protection. However, the main bactericidal substances of neutrophils are superoxide ions and lysosomal enzymes, whose excessive release will damage the surrounding cells and tissues and aggravate the inflammatory response. At the same time, the inflammatory cytokines produced and released by neutrophils in the process of phagocytosis of bacteria will also aggravate inflammation and promote the local inflammatory response of periodontal tissues, leading to damage and destruction of the periodontal tissues. IL-17A can also induce osteoblasts to secrete RANKL to participate in osteoclast differentiation, mediating alveolar bone absorption in patients with periodontitis (72). Yu et al. have shown that IL-17A plays a major protective role in bone loss in *Porphyromonas gingivalis* induced periodontitis, although a large number of researches have shown that IL-17A is closely related to bone erosion in rheumatoid arthritis (78). In addition, IL-17A can also act synergistically with IL-1 and TNF-α to induce gingival fibroblasts to produce MMP-1 and MMP-3, which plays an important role in the tissue destruction in periodontitis (79). IL-17A can promote keratinocytes to secrete antimicrobial peptides and play a defensive role, but whether it plays a role in the progression of periodontitis has not been confirmed. In the mouse model of ectopic tracheal transplantation, IL-17A is involved in the pathogenesis of obliterative bronchiolitis by regulating M1 macrophage polarization (44), but whether IL-17A mediates periodontal tissues destruction by promoting M1 macrophage polarization and secreting inflammatory factors remains to be studied.

## Role of Il-17A in the Relationship Between Periodontitis and Rheumatoid Arthritis

Rheumatoid arthritis (RA) is a chronic inflammatory and autoimmune disease characterized by synovitis and hyperplasia, articular cartilage destruction and bone erosion. Its main manifestation is invasive arthritis caused by the production of anti-citrulline protein antibody (ACPA), eventually leading to joint swelling and pain, cartilage destruction, and joint deformity and loss of function. There are many similarities between periodontitis and RA in pathological and immunological characteristics, including increased inflammation and immune cell infiltration, similar cytokine profile, increased release of proinflammatory mediators, decreased release of anti-inflammatory mediators, and activation of NF-κB/RANKL signaling pathway (80). The close relationship between periodontitis and RA has been widely confirmed by many studies (81). RA patients had higher prevalence and severity of periodontitis than healthy controls (82). It is considered that the periodontal tissues in chronic inflammatory state of periodontitis patients are the sites of ACPA generation (83). The periodontal condition of RA patients treated with anti-rheumatic drugs was better than that of untreated RA patients (84–86), suggesting that periodontal treatment may be a non-drug approach to improve rheumatoid arthritis (87).

The similar cytokine profile of periodontitis and RA includes increased levels of TNF-α, IL-1, MMPs and PEG2, and low levels of tissue inhibitors of metalloproteinases (TIMP). Kaczyński et al. found that the level of IL-17A in saliva of patients with RA was higher than that of healthy controls, but lower than that of patients with periodontitis, while the level of IL-17A in saliva of RA patients treated with anti-rheumatic drugs was reduced (88). Gümüş et al. found that the concentration of IL-17A in serum of RA patients with periodontitis was significantly higher than that of periodontitis patients without systemic diseases (89). The above findings suggested that the change of IL-17A level is not limited to local pathological tissues but is systemic in both periodontitis and RA.

In the process of inflammation of RA, Th17 cells secrete IL-17A induce NETosis in neutrophils and promote the formation of neutrophil extracellular trap which significantly augmented inflammatory responses in RA-FLSs to maintain the inflammatory state (90). In the late stage of inflammation, IL-17A can prolong the survival time of RA-FLSs, which contributes to synovial hyperplasia (91). In the process of joint destruction, IL-17A can promote osteoclast differentiation to cause bone resorption (64), induce FLSs and neutrophils to secrete MMPs to degrade bone matrix through collagen degradation (70, 92), and stimulate the expressions of iNOS and COX-2 in chondrocytes to cause articular cartilage destruction (42).

## Role Of Il-17A in the Relationship Between Periodontitis and Psoriasis

Psoriasis (P_S_O) is an autoimmune disease characterized by skin lesions. Cases of PsO can be characterized by psoriasis vulgaris, and other subtypes or psoriatic arthritis (P_S_A). Psoriasis vulgaris is characterized by scaly erythema or patches of which the boundary is clear and the distribution is limited or extensive. The relationship between periodontitis and PsO has been confirmed by many clinical studies. Zhang et al. suggested that periodontitis was positively correlated with psoriasis (93). Dalmády et al. proposed that periodontitis may play a direct or indirect role in the occurrence or exacerbation of PsO through the immunomodulatory effect of oral microbiota, and may affect the efficacy of anti-PsO treatment (94). Periodontal basic treatment can reduce the severity of PsO while improving periodontal status and reducing the expression level of inflammatory factors in saliva (95). Treatment of PsO with TNF-α inhibitors can also slow down the progression of periodontitis (96).

Psoriasis is a chronic inflammatory skin disease characterized by keratinocyte proliferation and accumulation of immune cells. PsO was previously thought to be a Th1 cell-mediated disease, but it is now believed that IL-23/IL-17 axis regulates the inflammatory progression of PsO. IL-17A can stimulate keratinocytes to produce antimicrobial peptides, chemokines and proliferative cytokines, thus inducing keratinocytes proliferation (97). In addition, IL-22, which has a protective effect on non-psoriatic skin, collaboratively aggravates psoriatic lesions with IL-17A in patients with PsO (98). Jiménez et al. found that the total concentrations of IL-17A, IL-22 and IL-23 in gingival crevicular fluid in patients with PsO and moderate or severe periodontitis were increased compared with those in PsO patients with mild periodontitis or without periodontitis (99). At present, the therapeutic drugs for PsO have been developed from TNF-α inhibitors to IL-17A inhibitors. The treatment of PsO with IL-17A inhibitors may also significantly slow down the occurrence and development of periodontitis, but there is still a lack of research evidence. IL-17A may be the link between PsO and periodontitis, but there is no direct evidence that there is a causal relationship between them, which needs to be further clarified.

## Role Of Il-17A in the Relationship Between Periodontitis and Diabetes Mellitus

Diabetes mellitus is classified as type I and type II. Type I diabetes mellitus, also known as insulin-dependent diabetes mellitus, is mainly caused by immune-mediated β cell defects in the pancreas resulting in absolute insulin deficiency. Type 2 diabetes mellitus (T2DM) is the most common type, also known as non-insulin-dependent diabetes mellitus, which is caused by insulin resistance and insulin production disorders. Insulin resistance is a reduction in tissue sensitivity to insulin. Insulin production disorder means that the levels of insulin molecule or its receptor are defective. As one of the sign of diabetes mellitus, hyperglycemia is closely related to the occurrence and severity of periodontitis, while periodontitis is known as the sixth complication of diabetes mellitus (100, 101). The immune inflammatory response caused by periodontitis is more likely to lead to insulin resistance, and the presence of periodontitis will increase the severity of diabetes mellitus (102). Therefore, periodontitis and diabetes mellitus show a bidirectional interaction and restriction relationship (103).

The level of IL-17A increased successively in saliva of healthy control group, T2DM patients and T2DM patients with chronic periodontitis, suggesting that the increase of IL-17A is one of the risk factors of chronic periodontitis in T2DM patients (104, 105). IL-17A is also a risk factor for diabetic retinopathy in T2DM (106, 107). However, the mechanism of IL-17A in the pathogenesis or progression of T2DM has not been elucidated.

## Role Of Il-17A in the Relationship Between Periodontitis and Other Systemic Chronic Inflammatory Diseases

Crohn’s disease (CD) is a chronic, nonspecific inflammatory disease characterized by local mucosal inflammation of the intestinal tract. The main symptoms are abdominal pain, diarrhea, and weight loss. CD and ulcerative colitis (UC) are collectively referred to as inflammatory bowel diseases (IBD) (108). Studies have shown that CD patients have an increased risk of periodontitis (109) and IBD is associated with more severe and extensive periodontitis, but more longitudinal studies are needed to support this (110, 111). Menegat et al. found that there was no difference in the expression level of IL-17A in gingival tissues of patients with CD combined with periodontitis and patients with UC combined with periodontitis, but the expression level of IL-17A in gingival tissues of these two patients was higher than that in intestinal mucosa (112). The role of IL-17A in the progression of IBD appears to be both favorable and unfavorable. Studies have shown that antibodies blocking IL-12 and IL-23 are effective in the treatment of IBD (113), but blocking IL-17A in CD has been found to be ineffective and has a higher incidence of adverse events. Both Secukinumab which targets IL-17A and Brodalumab which blocks IL-17RA are highly effective in PsO, but worse than placebo in CD (114, 115). The reason for the poor efficacy of IL-17A blocking on CD may be that IL-17A may drive mucosal inflammation and also contribute to the recovery and repair of intestinal mucosa after inflammation subsides, suggesting that attention should be paid to cytokine pluripotency and the complexity of regulatory cell network in intestinal mucosa (116).

Osteoporosis is a systemic and metabolic bone disease, which is mainly characterized by decreased bone mass, destruction of bone microstructure, increased bone fragility and decreased bone strength. It can be divided into 3 types including primary, secondary and idiopathic. Among them, primary osteoporosis is divided into postmenopausal osteoporosis and age-related osteoporosis. Many cross-sectional studies have confirmed the correlation between osteoporosis and periodontitis (117). Xu et al. showed that osteoporosis is closely related to the increased risk of occuring periodontitis (118). Women with postmenopausal osteoporosis treated with estrogen had a lower risk of severe periodontitis than untreated controls (119). At present, there are few studies on the correlation between IL-17A and osteoporosis. The concentration of IL-17A in the serum of periodontitis patients with osteoporosis was significantly higher than that of periodontitis patients without systemic diseases (89), suggesting that IL-17A may be related to the progression of osteoporosis. However, Goswami et al. found that IL-17RA^KO^ mice were more vulnerable to ovariectomy induced bone loss than the control group (120). The above results show that the IL-17A signal regulatory network is complex in the occurrence and development of osteoporosis.

Atherosclerosis is a chronic progressive process. Its pathological mechanism is that large and medium-sized muscular arteries and large elastic arteries are blocked by fibrotic and lipid plaques. More and more evidences show that inflammation caused by infection is significantly related to the increased risk of atherosclerosis and plays an important role in the initiation, occurrence and development of atherosclerosis (121, 122). Periodontitis is one of the important risk factors of atherosclerosis (123–125). Pavlic et al. detected periodontitis pathogens in carotid and coronary atherosclerotic plaques, including *Tannerella forsythia*, *Porphyromonas gingivalis*, *Actinobacillus actinomycetemcomitans* and *Prevotella intermedia*, suggesting that periodontitis pathogens can directly enter the atherosclerosis lesion area and may participate in the plaque formation process (126). Besides, *Porphyromonas gingivalis* can promote the oxidation of low-density lipoprotein (LDL) to oxidized LDL which causes the formation of atherosclerosis (127), and periodontal treatment can reduce oxidized LDL in plasma (128). Changes of immune response and inflammatory mediators caused by periodontitis may also promote the formation of atherosclerosis. In addition, Sasaki et al. recently found that tryptophan tRNA synthase produced by *Porphyromonas gingivalis* infected THP-1 cells is also associated with atherosclerosis (129). A large number of studies have shown that IL-17A is up-regulated in atherosclerosis, and its pathophysiological role in promoting atherosclerosis may be to increase plaque size and aggravate inflammation (130). The therapeutic drug digoxin for atherosclerosis is a direct antagonist of RORγt which is the main transcription factor of IL-17A. The treatment of atherosclerosis with digoxin can significantly reduce the circulating lipid, the expression level of IL-17A and the size of lesions in *Apoe -/-* mice, suggesting that reducing IL-17A is beneficial. However, digoxin is not exclusively targeted at IL-17A (131). However, Fang et al. demonstrated that propolis ethanol extract can inhibit the formation of atherosclerotic lesions in *Apoe -/-* mice fed a high-fat diet by increasing the level of IL-17A, which supports that IL-17A is an anti-atherosclerotic factor (132). Therefore, the exact role of IL-17A in the progression of atherosclerosis is still controversial.

IL-17A is also related to chronic inflammatory diseases such as multiple sclerosis, ankylosing spondylitis (AS), Behcet’s disease and active infected uveitis. However, the correlation between these diseases and periodontitis is not sufficiently proven, so it is not discussed in this work.

## Application Potential of Il-17A Targeted Therapy in Periodontitis and Related Systemic Chronic Inflammatory Diseases

At present, there are no clinical studies on IL-17A inhibitors for the treatment of periodontitis. In animal experiments, Pacheco recently found that continuous delivery of IL-17A neutralizing Antibodies in local periodontal region could limit inflammatory bone loss in experimental periodontitis mice (133). More research are needed to determine whether IL-17A inhibitors can be used topically to treat periodontitis and at what development stage of periodontitis that IL-17A inhibitors should be used.

Immune-mediated inflammatory diseases have traditionally been treated with glucocorticoids, non-steroidal anti-inflammatory drugs (NSAID), and disease modifying antirheumatic drugs (DMARD), which are effective in improving clinical symptoms and signs. However, intolerance, poor efficacy and severe adverse reactions have led to the need to develop alternative therapies (97). Monoclonal antibodies and fusion proteins, referred to as biological agents, were introduced in the early 1990s. TNF-α antagonists were first used in the treatment of clinical diseases, as shown in **Table 2**. However, the response and tolerance to different TNF antagonists vary among individuals. Some patients did not respond to the initial treatment of one or more drugs or lost response over time. Furthermore, patients treated with TNF-α antagonist were at increased risk of severe infection (134). Later, monoclonal antibodies against IL-23 and Il-12-IL-23 signal transduction appeared, as shown in **Table 2**.

**Table 2:** Anti-TNF-α, IL-12/23 p40, IL-23, IL-17A and IL-17RA agents approved by FDA.

Drug	Targets	Indications
Etanercept (Enbrel)	TNF-α	RA, polyJIA, plaque PsO, PsA, AS
Infliximab (Remicade)	TNF-α	RA, CD, UC, plaque PsO, PsA, AS
Adalimumab (Humira)	TNF-α	RA, CD, UC, plaque PsO, PsA, AS
Ustekinumab (Stelara)	IL-12/23 p40	plaque PsO, PsA, CD, UC
Guselkumab (Tremfya)	IL-23	plaque PsO
Tildrakizumab (Ilumya)	IL-23	plaque PsO
Risankizumab (ABBV-066)	IL-23	plaque PsO
Secukinumab (AIN457, Cosentyx)	IL-17A	plaque PsO, PsA, AS, nr-axSpA
Ixekizumab (LY24439821, Taltz)	IL-17A	plaque PsO, PsA, AS
Brodalumab (AMG827, Siliq)	IL-17RA	plaque PsO

Subsequent studies found that IL-17A plays an important role in the pathogenesis of RA, PsO, and AS. Compared with TNF-α and IL-12/23, IL-17A is closer to the downstream of the pathogenesis of disease, and the action target is relatively clear. Among IL-17A inhibitors (**Table 2**), Secukinumab (AIN457, Cosentyx) is approved by the US Food and Drug Administration (FDA) to treat plaque PsO, PsA, AS, nr-axSPA, Ixekizumab (LY2439821, Taltz) is approved to treat plaque PsO, PsA, and Brodalumab (AMG 827, Siliq) is approved for the treatment of plaque PsO. Bisides, there are new candidate drugs targeting IL-17A include CNTO 6785 targeting IL-17A, CoVA322 and Remtolumab (ABT-122) targeting both TNF-α and IL-17A, and Bimekizumab, Sonelokimab (M1095) and CJM112 targeting IL-17A/F (**Table 3**). Among them, FDA is currently reviewing the application of Bimekizumab for the treatment of adult patients with moderate to severe plaque PsO. Sonelokimab has shown excellent results in the clinical practice of phase IIb in the treatment of moderate to severe plaque PsO (146). At present, no IL-17A inhibitor has been approved by FDA for the treatment of RA. A phase III randomized, double-blind, active comparator- and placebo-controlled study have been showed that Secukinumab can improve the signs and symptoms of patients with active RA (149), while a phase II randomized study found that Ixekizumab improved the signs and symptoms of RA in patients who showed an inadequate response to TNF inhibitors (150). Further trials with IL-17A inhibitor in the treatment of RA are warranted.

**Table 3:** Novel drug candidates targeting IL-17A and IL-17A/F.

Drug	Targets	Possible Indications	References
CNTO 6785	IL-17A	RA, COPD	(135, 136)
COVA322	Bispecific TNF/IL-17A	plaque PsO	(137)
Remtolumab (ABT-122)	Bispecific TNF/IL-17A	RA, PsA	(138–141)
Bimekizumab	IL-17A/F	plaque PsO, PsA, AS	(142–145)
Sonelokimab	IL-17A/F	plaque PsO	(146)
CJM112	IL-17A/F	plaque PsO, hidradenitis suppurativa	(147, 148)

## Conclusions and Perspectives

**Figure 5:**
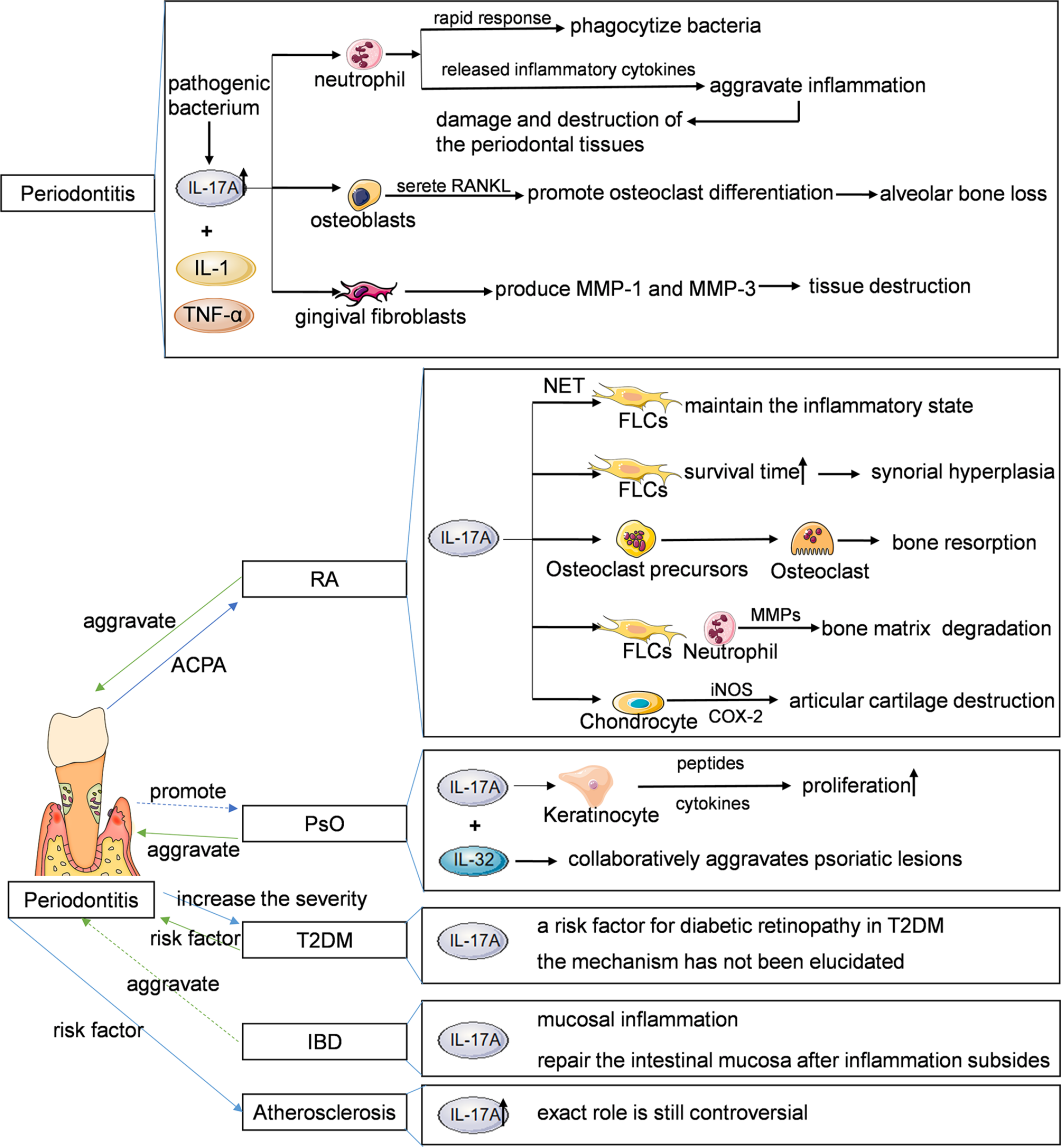
Main features of the IL-17-periodontitis-systemic chronic inflammatory diseases. IL, interleukin; RANKL, receptor activator of nuclear factor kappa B ligand; MMP, matrix metalloproteinase; NET, neutrophil extracellular trap; FLSs, fibroblast like synoviocytes; TNF-α, tumor necrosis factor-α; iNOS, inducible nitric oxide synthase; COX, cyclooxygenase; RA, rheumatoid arthritis; PSO, psoriasis; T2DM, type 2 diabetes mellitus; IBD, inflammatory bowel diseases.

IL-17A plays an important role in immune inflammatory responses in rheumatoid arthritis, psoriasis, diabetes, inflammatory bowel disease, atherosclerosis, and periodontitis (**Figure 5**). The patients with systemic chronic inflammatory diseases mentioned above are often accompanied by periodontitis concomitantly. Although the causal relationship between some of these diseases and periodontitis has not been fully confirmed and more researches are needed to provide new theoretical basis for clinical treatment, early prevention and treatment can reduce the risk of periodontitis. Similarly, early prevention and treatment of periodontitis is also conducive to the improvement of systemic chronic inflammatory diseases. At present, many studies have confirmed that blocking IL-17A can effectively treat psoriasis, rheumatoid arthritis and ankylosing spondylitis, but there are few studies on the use of IL-17A inhibitors in the treatment of periodontitis. This may be related to the fact that the mechanism of IL-17A in the progression of periodontitis has not been fully clarified, and IL-17A has both physiological and pathological effects, and IL-17A inhibitors have relatively great side effects. Further study on the mechanism of Th17/Treg balance and IL-17A signaling pathway in the occurrence and development of periodontitis and its specific association with systemic chronic inflammatory diseases will be helpful to provide more scientific and complete treatment strategies for the treatment of periodontitis and systemic diseases.

## Author Contributions

YF did the bibliography research and drafted the manuscript. HA revised the manuscript critically. All authors revised, read and approved the final manuscript.

## Funding

This work is supported by the Project funded by China Postdoctoral Science Foundation (No. 2021M703690), the GuangDong Basic and Applied Basic Research Foundation, China (No. 2021A1515010460 and 2021A1515111099) and the Science and Technology Projects in Guangzhou, China (No. 202102080157). HA is supported by the GuangDong Basic and Applied Basic Research Foundation, China (No. 2021A1515010460) and the Science and Technology Projects in Guangzhou, China (202102080157). YF is supported by the Project funded by China Postdoctoral Science Foundation (No. 2021M703690) and the GuangDong Basic and Applied Basic Research Foundation, China (No. 2021A1515111099).

## Conflict of Interest

The authors declare that the research was conducted in the absence of any commercial or financial relationships that could be construed as a potential conflict of interest.

## Publisher’s Note

All claims expressed in this article are solely those of the authors and do not necessarily represent those of their affiliated organizations, or those of the publisher, the editors and the reviewers. Any product that may be evaluated in this article, or claim that may be made by its manufacturer, is not guaranteed or endorsed by the publisher.

## Abbreviations

ACPA, anti-citrulline protein antibody; Act1, nuclear factor kappa B activator 1; AP-1, activator protein-1; AS, ankylosing spondylitis; CD, Crohn’s disease; C/EBP, CCAAT/enhancer binding protein; COL2A1, collagen type II alpha 1; COPD, chronic obstructive pulmoriary disease; COX, cyclooxygenase; CTL, cytotoxic T lymphocytes; CXCL, CXC chemokine ligand; DC, dendritic cells; DMARD, disease modifying antirheumatic drugs; ERK, extracellular signal-regulated kinase; FGF, fibroblast growth factor; FLSs, fibroblast like synoviocytes; G-CSF, granulocyte-colony stimulating factor; GM-CSF, granulocyte macrophage-colony stimulating factor; IBD, inflammatory bowel diseases; IFN-γ, Interferon-γ; IL, interleukin; IL-17R, interleukin-17 receptor; ILC3s, group 3 innate lymphoid cells; iNOS, inducible nitric oxide synthase; JNK, c-Jun N-terminal kinase; LDL, low-density lipoprotein; LL-37, antibacterial protein LL-37 amide; MAIT, mucosal associated invariant T; MCP-1, monocyte chemoattractant protein 1; MMP, matrix metalloproteinase; NF-κB, nuclear factor kappa B; NK, natural killer; NSAID, non-steroidal anti-inflammatory drugs; PDGF, platelet derived growth factor; P_S_O, psoriasis; P_S_A, psoriatic arthritis; RA, rheumatoid arthritis; RANK, receptor activator of nuclear factor kappa B; RANKL, receptor activator of nuclear factor kappa B ligand; sFRP, secreted frizzled-related protein; TAK, TGF-β-activated kinase; TGF-β, transforming growth factor-β; Th, helper T; TIMP, tissue inhibitor of matrix metalloproteinases; TLR-4, toll like receptor 4; Tn, neutral regulatory T; TNF-α, tumor necrosis factor-α; T2DM, type 2 diabetes mellitus; UC, ulcerative colitis; Wnt, wingless-type MMTV integration site family.
